# Neuroinflammation and COVID-19 Ischemic Stroke Recovery—Evolving Evidence for the Mediating Roles of the ACE2/Angiotensin-(1–7)/Mas Receptor Axis and NLRP3 Inflammasome

**DOI:** 10.3390/ijms23063085

**Published:** 2022-03-13

**Authors:** Che Mohd Nasril Che Mohd Nassir, Mohd K. I. Zolkefley, Muhammad Danial Ramli, Haziq Hazman Norman, Hafizah Abdul Hamid, Muzaimi Mustapha

**Affiliations:** 1 Department of Neurosciences, School of Medical Sciences, Universiti Sains Malaysia, Kubang Kerian 16150, Kelantan, Malaysia; 2 Faculty of Industrial Sciences and Technology, Universiti Malaysia Pahang, Lebuhraya Tun Razak, Gambang Kuantan 26300, Pahang, Malaysia; khairulizamil@yahoo.com; 3 Department of Diagnostic and Allied Health Science, Management and Science University (MSU), Shah Alam 40100, Selangor, Malaysia; muhammaddanialramli33@gmail.com; 4 Anatomy Unit, International Medical School (IMS), Management and Science University (MSU), Shah Alam 40100, Selangor, Malaysia; manziq91@gmail.com; 5 Department of Human Anatomy, Faculty of Medicine and Health Sciences, Universiti Putra Malaysia, Serdang 43400, Selangor, Malaysia; a_hafizah@upm.edu.my; 6 Hospital Universiti Sains Malaysia, Jalan Raja Perempuan Zainab II, Kubang Kerian 16150, Kelantan, Malaysia

**Keywords:** ischemic stroke, COVID-19, NLRP3 inflammasome, ACE2, neurorehabilitation

## Abstract

Cerebrovascular events, notably acute ischemic strokes (AIS), have been reported in the setting of novel coronavirus disease (COVID-19) infection. Commonly regarded as cryptogenic, to date, the etiology is thought to be multifactorial and remains obscure; it is linked either to a direct viral invasion or to an indirect virus-induced prothrombotic state, with or without the presence of conventional cerebrovascular risk factors. In addition, patients are at a greater risk of developing long-term negative sequelae, i.e., long-COVID-related neurological problems, when compared to non-COVID-19 stroke patients. Central to the underlying neurobiology of stroke recovery in the context of COVID-19 infection is reduced angiotensin-converting enzyme 2 (ACE2) expression, which is known to lead to thrombo-inflammation and ACE2/angiotensin-(1–7)/mitochondrial assembly receptor (MasR) (ACE2/Ang-(1-7)/MasR) axis inhibition. Moreover, after AIS, the activated nucleotide-binding oligomerization domain (NOD)-like receptor (NLR) family pyrin domain-containing 3 (NLRP3) inflammasome may heighten the production of numerous proinflammatory cytokines, mediating neuro-glial cell dysfunction, ultimately leading to nerve-cell death. Therefore, potential neuroprotective therapies targeting the molecular mechanisms of the aforementioned mediators may help to inform rehabilitation strategies to improve brain reorganization (i.e., neuro-gliogenesis and synaptogenesis) and secondary prevention among AIS patients with or without COVID-19. Therefore, this narrative review aims to evaluate the mediating role of the ACE2/Ang- (1-7)/MasR axis and NLRP3 inflammasome in COVID-19-mediated AIS, as well as the prospects of these neuroinflammation mediators for brain repair and in secondary prevention strategies against AIS in stroke rehabilitation.

## 1. Introduction

The current pandemic of the highly contagious novel coronavirus disease (COVID-19) is continuing to impose a significant public health challenge worldwide. Aside from being a primary respiratory disease, numerous complications associated with the disease are being recognized, including acute ischemic strokes (AIS). Moreover, patients with COVID-19 who develop AIS are at a higher risk of a negative functional outcome compared to non-COVID-19 AIS patients [1]. The pathogen that is responsible for this devastating event is the severe acute respiratory syndrome coronavirus 2 (SARS-CoV-2)—an enveloped, single-stranded, positive-sense ribonucleic acid (RNA) virus of the Coronaviridae family with a phospholipid bilayer capsule containing spike proteins [2]. SARS-CoV-2 gains access to the body through the interaction of its spike (S) protein and angiotensin-converting enzyme 2 (ACE2), which is found in many tissues, including the brain. This, in turn, provokes an immediate immunological cascade of inflammatory responses that affects individual cells and triggers the subsequent thrombo-inflammation in the vascular system [3].

This is followed by the development of endothelial dysfunction and/or endotheliitis, which is primarily due to direct viral infection and the downregulation of ACE2 receptors and ACE2/angiotensin (1–7)/mitochondrial assembly receptor (MasR) (ACE2/Ang-(1-7)/MasR) axis [4]. In parallel, there is also the ongoing production of pro-inflammatory agents (i.e., cytokines such as interleukins (IL-10, -8, -6, and -17), chemokines, granulocytes colony stimulating factors (G-CSF), and tumor necrosis factor-α (TNF-α)) due to viral infection and the activation of transcription factors, such as inflammatory complex, nucleotide-binding oligomerization domain (NOD)-like receptor (NLR) family pyrin domain-containing 3 (NLRP3) inflammasome, and nuclear factor kappa B (NF-κB), which activate the release of pathogen-associated molecular patterns [5].

In the brain, these responses subsequently heighten the production of numerous pro-inflammatory cytokines, mediating neuro-glial cell dysfunction, ultimately leading to nerve-cell death [3]. Thus, neurorestorative opportunities manifest themselves through the understanding of the molecular mechanisms of the aforementioned mediators, which may inform rehabilitation strategies in terms of neuro-gliogenesis and synaptogenesis among AIS patients with or without COVID-19 intercurrent infection. This narrative review aims to evaluate the mediating role of the ACE2/Ang-(1-7)/MasR axis and NLRP3 inflammasome in COVID-19-mediated AIS and to highlight the prospects of these neuroinflammation mediators for brain repair and in secondary prevention strategies against AIS in stroke rehabilitation.

### Literature Search Strategy

The literature search strategy included the use of search engines and online databases using specific keywords combinations. Several online databases were used, including Google Scholar, PubMed, the ISI Web of Knowledge, and SCOPUS. The articles were restricted to those in the English language only. Furthermore, free full-text and open access articles, such as original research papers, narrative and systematic reviews, meta-analyses, randomized clinical trials, and cohort studies were selected. The literature search was based on the following keywords: COVID-19, neuroinflammation, ischemic stroke, ACE2/Ang-(1-7)/MasR axis, NLRP3 Inflammasome, Long-COVID syndrome, and stroke recovery or neurorehabilitation.

## 2. COVID-19 and the Incidence of Ischemic Stroke

To date, there are over 320 million confirmed cases of infection due to SARS-CoV-2 globally, with over 5 million cases of mortality, along with the administration of over 9 billion total COVID-19 vaccine doses (Johns Hopkins University & Medicine, Coronavirus Resource Center, https://coronavirus.jhu.edu, accessed on 16 January 2022). Moreover, increasing evidence has confirmed that COVID-19 and its complications are associated with thrombo-inflammatory events, including cerebrovascular disease (CVD) and neurological diseases, such as AIS and intracerebral hemorrhage [6,7,8,9].

### 2.1. COVID-19: Acute to Post-Acute Infection

The original outbreak of the COVID-19 infection can be traced back to Wuhan, in the Hubei province of the Republic of China, in late 2019 [10]. A recent report from genomic studies suggested that the genetic sources of α- and β-coronaviruses (CoVs) were derived from bats and rodents, and from an avian host in the case of δ- and γ-CoVs [11]. Moreover, about seven human CoVs have been recognized that potentially cause mild-to-severe diseases (including neurological disease) in various animal species such as cats, camels, and cows (including cattle) [12]. Alarmingly, recent scientific evidence has shown that these CoVs are accountable for nearly 10% of acute respiratory infections [11,13], and about 15% of individuals infected by members of the genus β-CoVs (i.e., SARS-CoV-2 and Middle East respiratory syndrome coronavirus (MERS-CoVs)) will develop severe disease, among whom 6% become critically ill [11,14]. On the other hand, the acute, subacute, and post-acute/long-term effects of SARS-CoV-2 infection (or COVID-19) could potentially lead to respiratory failure and/or multiple organ dysfunction (MODS) [15].

Moreover, the involvement of the respiratory and/or gastrointestinal systems during acute SARS-CoV-2 infection may give rise to symptoms such as fatigue, chest pain, shortness of breath, fever, dyspnea, diarrhea, nausea, arthralgia, vomiting, cognitive disturbance, and a reduced quality of life [16,17,18,19]. Such a wide spectrum of symptomatology is reflected through the underlying cellular damage and heighten the thrombo-inflammation (i.e., hypercoagulation/coagulopathy and cytokine storm-hyperinflammation) induced by SARS-CoV-2 infection [20,21]. Previous studies have also suggested that cytokine storm is the major cause of morbidity in patients infected with human CoVs (i.e., SARS-CoVs and MERS-CoVs) [22], and previous survivors of infection with these viruses (i.e., SARS in 2003 and MERS in 2012) have shown similarly persistent symptoms. this further reinforces the concern over significant COVID-19 long-term sequelae (from acute to post-acute to long COVID) [23,24,25,26].

The definition and pathophysiological mechanisms of post-acute and long-COVID remain contentious. However, it is generally accepted that post-acute COVID-19 implies the persistence of symptoms and/or the onset of sequelae after 3 to 4 weeks of acute infections [27]. This is because the replication-competent SARS-CoV-2 is not isolated after 3 weeks of acute infection [28]. Meanwhile, a recent review has suggested that post-acute COVID-19 involves persistent symptom/s and/or delayed or long-term complications of SARS-CoV-2 infection beyond 4 weeks from the onset of symptoms [29]. Furthermore, post-acute COVID-19 can be further categorized into two stages, namely, sub-acute (i.e., the ongoing symptomatic COVID-19, within 4–12 weeks) and post-acute/chronic COVID-19 and/or long-COVID syndrome (i.e., the persistence or reoccurrence of symptoms beyond 12 weeks from the first acute infection) [30]. Several potential pathophysiological mechanisms have been proposed for post-acute COVID-19, including virus-specific pathophysiological changes, heightened thrombo-inflammation, and the expected sequelae of post-critical illness [29].

Furthermore, multiple molecular risk factors related to SARS-CoV-2 and the occurrence of COVID-19 (irrespective of variants of concern, VOCs) could mediate extreme surges and the untimely onset of AIS [31,32]. These risk factors include generalized hypercoagulability, poorly regulated immune response leading to cytokine storm (or cytokine release syndrome), endothelial cell (EC) damage (i.e., endothelial dysfunction or endotheliitis) leading to elevated levels of microthrombogenic extracellular-derived circulating microparticles, increased thrombo-inflammation, renin-angiotensin system (RAS) dysregulation, and direct cytotoxic effects on the nervous system related to the ACE2 receptor uptake of SARS-CoV-2 viruses [33,34].

### 2.2. COVID-19-Mediated Ischemic Stroke

Given that COVID-19 can cause MODS involving the kidney, heart, and brain, it is increasingly regarded as a vascular disease associated with thrombo-inflammatory events that mainly affect vascular ECs [35,36]. Autopsies of multiple organs studied in patients with SARS-CoV-2 infection demonstrated microvascular and macrovascular thrombosis in the arteries, arterioles, capillary bed, and venules, consisting of platelets, fibrin, erythrocytes, and leukocytes, as well as cellular-derived microparticle deposition, supporting the fact that, rather than merely infecting the airway, SARS-CoV-2 manifests as coagulopathy and vasculopathy [33,34]. The extent of the nervous system’s involvement in COVID-19 infection is common from early acute infection (i.e., symptoms such as anosmia, dizziness, and headaches) to post-acute infection or the widely used term ‘brain-fog’ and fatigue, with the persistence of symptoms and multiple neurological manifestations [37,38]. Therefore, it is plausible that COVID-19 can mediate ischemic stroke [9].

In fact, it has been reported that COVID-19 patients possess higher odds (up to seven-fold) of developing ischemic stroke and are likely to be more severely affected by higher morbidity and mortality compared to non-COVID-19 ischemic stroke patients [9,39]. Whilst most ischemic stroke cases occur in those with other comorbidities and cardio-cerebrovascular risk factors [40], it can also occur in patients without comorbidities or even at a younger age (≤50 years of age) [41]. Even healthy and/or medically asymptomatic individuals are at risk of developing ischemic stroke in the context of COVID-19 infection [33,42].

Additionally, most cases of COVID-19-mediated ischemic stroke were reported as cryptogenic or embolic stroke of undetermined significance (ESUS), i.e., a subtype of stroke caused by an undiagnosed cardioembolic source, whilst most COVID-19-positive patients were asymptomatic prior to their ischemic event [43,44]. Therefore, understanding the pathophysiological mechanisms and the assessment of the long-term risk of ischemic stroke, specifically the cryptogenic subtype, may provide crucial information for future risk stratification and brain repair/rehabilitation strategies for the treatment of COVID-19-mediated ischemic stroke.

### 2.3. Pathophysiological Mechanism of COVID-19-Mediated Ischemic Stroke

The exact mechanism underlying COVID-19-mediated cardio-cerebrovascular disease (i.e., ischemic stroke) is still under extensive investigation. It is further complicated by cofounding variables and/or comorbidities, such as the presence of conventional vascular risk factors (e.g., type-2 diabetes mellitus, hypertension, and coronary heart disease) prior to SARS-CoV-2 infection among these patients [40,45]. Although the pathophysiological mechanisms of COVID-19-mediated ischemic stroke are understudied, they are comparable to the pathophysiology of AIS unrelated to COVID-19 infection [46].

Prior to COVID-19-mediated ischemic stroke, the viruses primarily target the ACE2 receptors that are present in various cell surfaces in multiple organs/systems. In the case of COVID-19, the cells in the respiratory system (e.g., alveolar pneumocytes) are the main targets, provoking an immediate inflammatory response in the form of an immunological cascade of that affects individual cells and subsequent thrombo-inflammation in the vascular system [3]. This is followed by the activation of ECs and subsequent endothelial dysfunction and/or endotheliitis and ACE2 receptor downregulation. Furthermore, the ongoing production of pro-inflammatory agents (i.e., cytokines, such as IL-10, -8, -6, and -17, chemokines, G-CSF, and TNF-α) and the activation of transcription factors, such as NLRP3 inflammasome and NF-κB, consequently activates the release of pathogen-associated molecular patterns [5]. Interestingly, acute strokes share similar responses i.e., bioenergetic failure and hypoxia secondary to ischemia, followed by damage-associated molecular patterns (DAMPs). Moreover, similar transcription factors are also activated and remain active throughout the early stages of cerebral brain ischemia or infarction, provoking chronic thrombo-inflammation and the suppression of immunological recovery [5,47].

**Figure 1:**
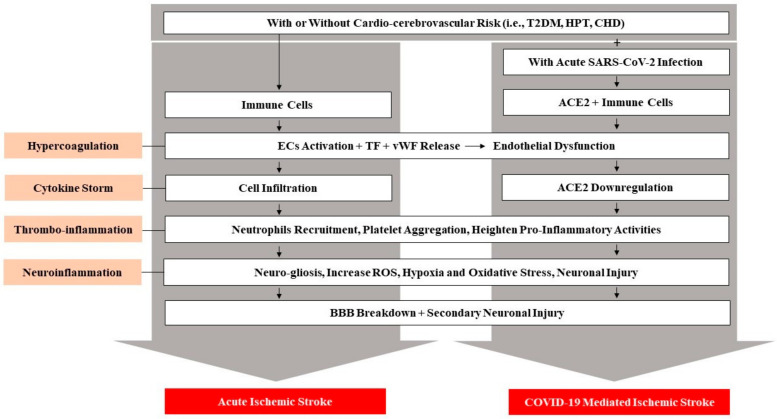
Pathophysiological mechanism of COVID-19-mediated ischemic stroke and its common or shared features with that of AIS. ACE2, angiotensin-converting enzyme 2; CHD, coronary heart disease; ECs, endothelial cells; HPT, hypertension; ROS, reactive oxygen species; T2DM, type 2 diabetes mellitus; TF, tissue factors; vWF, von Willebrand factor.

In the setting of acute COVID-19, prolonged alterations could spur imbalance in the RAS system, including ACE2 pathway downregulation, which may lead to end organ dysfunction. For example, the activation of the innate immune response in the vascular system induces the activation and recruitment of monocytes, macrophages, effector T cells, and neutrophils to the alveoli. These then intensify the release of pro-thrombotic agents, such as tissue factors (TF) and von-Willebrand factor (vWF), leading to a prothrombotic state and/or hypercoagulation [3]. Furthermore, the increase in the recruitment of intravascular neutrophils in the lungs and the microglia activation in the brain further influences the production of pro-inflammatory cytokines. Alarmingly, the excess pro-thrombo-inflammatory agents (e.g., cytokine storm and hypercoagulation) may circulate from peripheral circulation to the cerebral micro-vasculature. This can subsequently lead to neurovascular endotheliitis, blood–brain barrier (BBB) breakdown, cerebral micro-thrombosis, and thrombo-embolism [3]. Therefore, it is plausible that these pathophysiological mechanisms may disrupt the neuro-gliovascular unit. Figure 1 summarizes the pathophysiological mechanism of COVID-19-mediated ischemic stroke and the features it shares with that of AIS.

Moreover, a recent meta-analysis also suggested that individuals infected with COVID-19 who then developed AIS may be predisposed to anxiety and depression [48]. A previous study suggested that heightened inflammation (in AIS) is involved in the propagation of post-stroke depression [49]. Additionally, it has been postulated that anxiety-induced changes in the hypothalamic–pituitary adrenal system due to COVID-19 may also reinforce the imbalance between sympathetic and parasympathetic activity, wherein a group of individuals (either with or without prior ischemic stroke) with comorbidities and/or higher cardio-cerebrovascular risk (i.e., with maladaptive immune system and compromised vascular systems) is expected to demonstrate a post/long-COVID-19 hyperimmune response [5]. Therefore, recent reviews have provided several common post-acute to long-COVID-19 symptoms associated with impaired neurological systems (including prior ischemic stroke). Among them are fatigue, joint pain, chest pain, dyspnea, cough, insomnia, fevers, changes in sense of taste and smell, headaches, myalgia and weakness, neurocognitive difficulties, and depression [29]. Hence, understanding the underlying molecular and pathophysiological mechanism of COVID-19-mediated ischemic stroke may provide clues as to COVID-19 ischemic stroke recovery and brain repair. This, in turn, may provide new insights into the management of post-acute to long-COVID-19 neurological syndrome.

## 3. Role of ACE2/Ang-(1-7)/MasR Axis in COVID-19-Mediated Ischemic Stroke

**Figure 2:**
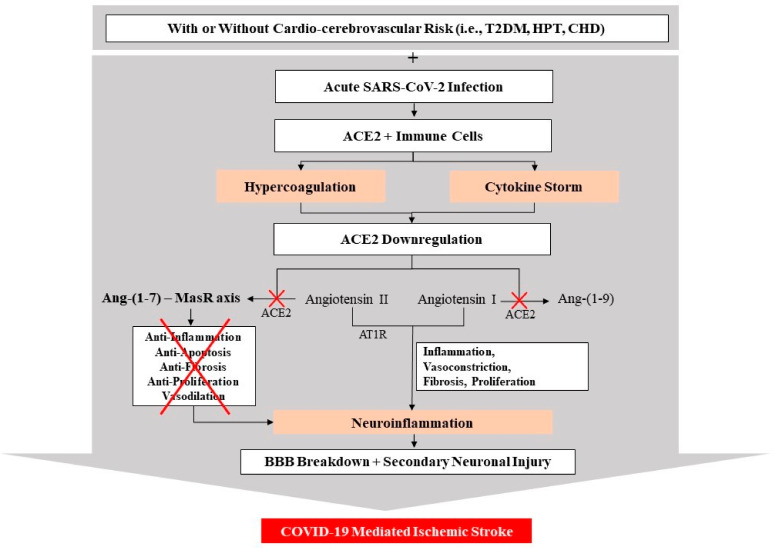
Possible mechanism of COVID-19-mediated ischemic stroke due to SARS-CoV-2-infection-mediated downregulation of angiotensin-converting enzyme 2 (ACE2) from renin angiotensin system (RAS). Reduced expression of ACE2 inhibits the conversion of angiotensin II to Ang-(1-7) and of angiotensin 1 to Ang-(1-9). Reduced Ang-(1-7) activity and its axis with mitochondrial assembly receptor (MasR) interfere with the anti-inflammatory, anti-apoptosis, anti-fibrosis, and vasodilation effect, thereby increasing blood–brain barrier (BBB) permeability and damage. Furthermore, the overactivation of angiotensin II binds to its angiotensin II type 1 receptor (AT1R), promoting further inflammation, vasoconstriction, fibrosis, and proliferation, thereby increasing secondary (2°) neuro-glial cell injury, leading to neuroinflammation and, finally, brain ischemia. CHD, coronary heart disease; HPT, hypertension; T2DM, type 2 diabetes mellitus.

As discussed, one of the possible mechanisms of endothelial dysfunction and/or endotheliitis in COVID-19 patients is a disrupted RAS, including the downregulation of ACE2 receptors, found in vascular ECs [4]. An integral part of RAS is the homolog ACE and ACE2 [4]. The RAS system consists of the classical arm and renin/ACE/angiotensin II/angiotensin II type 1 receptor (AT1R) axis, which plays pathophysiological roles in MODS, including the brain. Moreover, the ACE2/Ang-(1-7)/MasR axis has been recognized as a counter-regulator of angiotensin II [50]. Additionally, ACE2 impedes the action of ACE by turning angiotensin II into angiotensin (1–7) (i.e., an anti-inflammatory and a vasodilator molecule) and transforming angiotensin I into angiotensin (1–9), which is further converted to angiotensin (1–7) [51]. Angiotensins (1–7) moderate their effect through the MasR. Consequently, the binding of SARS-CoV-2 to ACE2 instigates receptor endocytosis, leading to the depletion or downregulation of the “protective” endothelial ACE2. This downregulation of ACE2 creates an imbalance between ACE and ACE2, thus elevating the pathological activation of the ACE/Ang II/AT1R axis. In turn, this leads to Ang II-mediated vasoconstriction and reduces Ang (1–7)-mediated vasodilation [4]. Besides ACE2 downregulation, the interrupted balance in the demand of ACE and angiotensin II with an increment of proinflammatory disposition and endothelial damage contributes to the pathogenesis of ischemic stroke [4]. Figure 2 summarizes the possible mechanism of action related to COVID-19 and ACE2 downregulation.

Moreover, it has also been demonstrated that the downregulation of ACE2 may metabolize its biological substrates, such as Des-arg9-BK (DABK), through the activation of DABK/bradykinin receptor B1 (BKB1R) axis signaling. This triggers inflammation through the release of several pro-inflammatory agents, such as chemokines and tumor necrotic factors, which leads to vascular dysfunction and increased neutrophil infiltration [52].

In the case of COVID-19, it has been proposed that direct SARS-CoV-2 invasion of the brain may occur through two potential channels. The first is hematogenous spread to the cerebral circulation due to systemic distribution; the second is transmission through the olfactory epithelium via the cribriform plate to the olfactory bulb [53]. As SARS-CoV-2 is present in general systemic circulation, stagnant cerebral microcirculation increases the reciprocity between ACE2 and the spike protein of SARS-CoV-2 on cerebral ECs [54]. Viral proliferation and release from ECs may lead to endothelial and BBB damage, allowing viral access to the brain parenchyma [54]. Additionally, ACE2 receptors were discovered on neuro-glial cells, making them a probable target for the spread of SARS-CoV-2 infection [55].

### Neuroprotective Mechanisms of the ACE2/Ang-(1-7)/MasR Axis in Post-Ischemic Stroke

The discovery of the favorable neuroprotective potential of the ACE2/Ang-(1-7)/MasR axis in AIS has attracted a growing body of research aiming expand the characterization of its mechanisms of action. The traditional view of the RAS has been further expanded with the discovery of an alternative pathway for angiotensin peptide signaling and metabolism, in which angiotensin II is hydrolyzed by ACE2 to form angiotensin-(1-7) [56]. This heptapeptide binds to and activates a unique G-protein-coupled receptor (GPCR), known as Mas, to exert effects that are largely in direct opposition to those of angiotensin II type 1 receptor (AT1R) activation [56]. To date, converging evidence has shown several perspectives on the neuroprotective actions of angiotensin-(1-7) in stroke, with specific mechanisms that may account for its protective effects, including anti-inflammatory, antioxidant, angiogenic, and vasodilatory effects, and the role of altered kinase–phosphatase signaling. Crosstalk between Mas and other receptors, such as bradykinin receptors (i.e., BKB1R) and angiotensin II type 2 receptors, were also explored [56].

Following an ischemic stroke, the neuro-glial cells exhibit all the elements of the ACE2/Ang-(1-7)/MasR axis [57]. Nevertheless, the expression of AT1R was shown to be reduced in the cerebral cortex after transient middle-cerebral artery occlusion (tMCAO) [58], but elevated in the rostral ventrolateral medulla (RVLM) [59]. For example, Lu et al., in their animal study, showed that ischemic stroke itself causes the increased levels of ACE2 and angiotensin-(1-7). Furthermore, they also reported that ischemic stroke resulted in the appearance of elevated levels of angiotensin-(1-7), ACE2, and Mas in samples of rat cortex in the 48h following ischemic stroke [60]. Another study showed that within the RVLM, ischemic stroke led to a decrease in Ang-(1-7) levels for up to three days post-stroke and an initial reduction in Mas one day after ischemic stroke, followed by significant increments after three and seven days of MCAO in rats [59]. The gene array results from this study also indicated an increase in ACE2 expression one day post-stroke. Another study, based on human stroke patients, revealed that serum ACE2 levels were notably elevated among cardioembolic strokes; the authors deduced that changes in ACE2 can be informative in the diagnosis and prognosis of stroke subtypes [61].

Moreover, the upregulation of the ACE2/Ang-(1-7)/MasR axis pathway has been shown to have broad therapeutic potential in stroke [56,62]. For example, the counterbalancing of the ACE/Ang II/AT1R axis by the protective ACE2/Ang-(1-7)/MasR axis pathway led to neuroprotection in stroke models [56]. The overexpression of ACE2 led to neuroprotective effects and may aid in brain repair [63]. The intracerebroventricular infusion of an ACE2 activator notably reduced the size of cerebral infarct and neurological deficits [51]. A previous in vitro study also reported that MasR agonists (i.e., AVE 0991) exert direct protective effects on neuronal cells [64]. Furthermore, in a tMCAO rat stroke model, Kuipers et al. found that the administration of peptidase-resistant, lanthionine-stabilized angiotensin-(1-7) (cAng-(1-7)) clearly and notably improved motor functions, as evaluated by neuro-score, stepping test, and body swing test [65]. They also found that the sensory motor functions were sensitive to treatment with cAng-(1-7), evidenced by the forelimb placement test [65]. In summary, these data indicate that the improved neurorehabilitation of performance might result from enhanced MasR stimulation and the regulation of the ACE2/Ang-(1-7)/MasR axis.

In COVID-19-mediated ischemic stroke, where the protective effects of ACE2 are downregulated or depleted due to the interaction between S protein and SARS-CoV-2, the balance is tilted in favor of ACE and angiotensin II. Understanding the neuroprotective mechanism of the ACE2/Angiotensin-(1-7)/MasR axis in post-ischemic stroke and the modulation of this pathway by regulating the expression of the different specific biomarkers involved in both axes could open a new door for a potential therapeutic target to elicit neuroprotection i.e., improve neuro-gliogenesis and/or synaptogenesis for COVID-19-mediated ischemic stroke. Further evidence pertaining to the positive neuro-gliogenesis and/or synaptogenesis effect of the upregulation of ACE2/Angiotensin-(1-7)/MasR axis are discussed in a later section.

## 4. Role of NLRP3 Inflammasome in COVID-19-Mediated Ischemic Stroke

Another potential mediator of ischemic stroke that has recently gained attention is the NLRP3 inflammasome, a member of the NLR family of inherent immune cell sensors. Through innate immune-cell sensors, such as pattern recognition receptors (PPRs), it has been suggested that NLRP3 inflammasome may potentially detect pathogens or viral invasion and cell-to-tissue damage [66]. Moreover, PPRs are also involved in the activation of NF-κB and mitogen-activated protein kinase (MAPK) pathways, thereby elevating the transcription of genes that encode proteins related to the NLRP3 inflammasome. Alarmingly, an increase in the activity of NLRP3 inflammasome has been associated with ischemic stroke because the activated NLRP3 inflammasome is thought to mediate neuro-glial cell aberration or dysfunction and cerebral edema, leading to neuro-glial cell death by producing various pro-inflammatory agents (i.e., cytokines) [66]. To date, its translational merit for clinical treatment and therapeutic strategies for ischemic stroke remains largely unexplored, especially in targeting their pathophysiological mechanism, even at the molecular level. Therefore, further understanding of the role of NLRP3 inflammasome may open a new window for potential approaches to ischemic stroke therapy and brain repair/rehabilitation.

### 4.1. NLRP3 Inflammasome and Its Mechanism of Activation

NLRP3 inflammasome is the most widely studied inflammatory complex that is associated with aseptic inflammation [67]. It has been reported to be involved in multiple inflammatory, infectious, and immune diseases [68]. Structurally, NLRP3 inflammasome has three protein subunits, including effector pro-caspase subunit, adaptor protein–apoptosis-associated speck-like protein containing a CARD (ASC) subunit, and the NLRP3 receptor subunit [69]. On the other hand, the NLRP3 receptors consist of three main domains: N-terminal pyrin (PYD) domain (which is important for NLR protein to bind with adaptor protein ASC), C-terminal leucine-rich repeat (LRR) domain, and NAIP, CIITA, HETE-E, and TP1 (NACHT) domain (which is important for the formation of NLRP3 receptor and act as central core of the inflammasome) [70]. Furthermore, the inflammasome may serve as a molecular agent for the activation of caspase-1 and the modulation of innate immune response [71], where caspase-1 regulates the protein cleaving and pruning of pro-IL-1β and pro-IL-18 into functional pro-inflammatory cytokines, i.e., IL-1β and IL-1, respectively, before being released into the extracellular space [72].

There are several mechanisms involved in the activation of the NLRP3 inflammasome. In general, reduced concentrations of intracellular potassium ion (K^+^) [73], increased concentrations of intracellular calcium ion (Ca^2+^) [74], and the uncontrolled production of reactive oxygen species (ROS) [75] are the main known mediators of NLRP3 inflammasome activation. In short, following brain ischemia, the respective increments of K^+^ (outflow) and Ca^2+^ (inflow) are mediated by heightened adenosine triphosphate (ATP) concentrations, which promote the activation of purinergic ligand-gated ion channel 7 receptor (P2 × 7R) [76]. Meanwhile, following hypoxia and ischemia, mitochondrial dysfunction and oxygen deprivation lead to the overproduction of ROS [77], supporting the notion that mitochondria are important for ROS-mediated NLRP3 inflammasome activation [75]. Moreover, during ischemia, the stressed endoplasmic reticulum (ER) may induce the further formation of ROS through nicotinamide adenine dinucleotide phosphate (NADPH) oxidase (NOX) [78]. An elevated amount of ROS in the ER also led to mitochondrial Ca^2+^ deposition and further aggravated mitochondrial damage [79]. In fact, oxidized mitochondrial DNA can also lead to the excitation and activation of the NLRP3 receptor, in parallel with the role of mitochondria in the signal transduction of the NLRP3 inflammasome [80]. Recent evidence also suggested that double-stranded RNA also activated the NLRP3 inflammasome through protein kinase R (PKR) following reduced intracellular K^+^, increased intracellular Ca^2+^ levels, and increased ROS [81].

**Figure 3:**
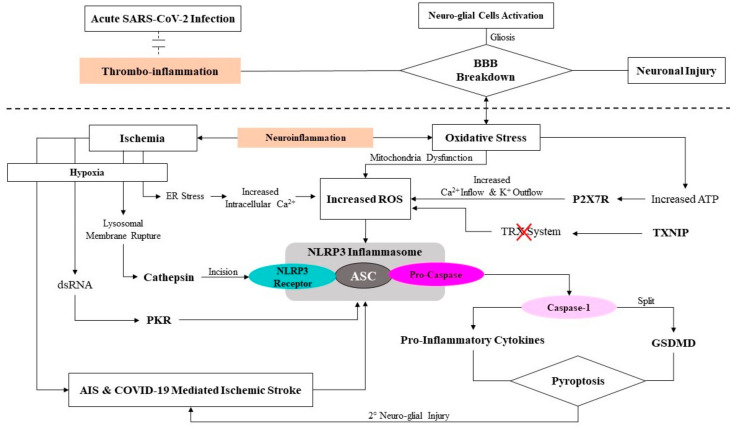
Brief mechanism of NLRP3 inflammasome activation and its role in acute ischemic stroke (AIS) and COVID-19-mediated ischemic stroke. Upon viral infection and subsequent increases in thrombo-inflammation and blood–brain barrier (BBB) breakdown, the heightened oxidative stress promotes higher activity of adenosine triphosphate (ATP), activating purinergic ligand-gated ion channel 7 receptor (P2X7R), thereby increasing calcium ion (Ca^2+^) inflow and potassium ion (K^+^) outflow. These, in turn, increase reactive oxygen species (ROS) production. A higher production of ROS can also be induced by oxidative-stress-mediated mitochondrial dysfunction and the inhibition of the thioredoxin (TRX) system by TRX-interacting protein (TRXNIP). The increased ROS then activates the NLRP3 inflammasome. Furthermore, increased ROS-mediated NLRP3 inflammasome can also be mediated by ischemic mediated endoplasmic reticulum (ER) stress, which increases intracellular Ca^2+^ lysosomal membrane rupture, which in turn activates NLRP3 receptor incision through cathepsin and activated protein kinase R (PKR) by double-stranded RNA. The activated NLRP3 inflammasome promotes the pro-caspase self-cleavage into caspase-1; next, the caspase-1 lyses and activates gasdermin-D-mediated cell death (GSDMD) and pro-inflammatory cytokines (i.e., interleukin-18 and -1β), leading to neuro-glial cell death or pyroptosis, inducing or worsening AIS or COVID-19-mediated ischemic stroke. ASC, apoptosis-associated speck-like protein containing a CARD subunit.

Additionally, a mounting body of evidence suggests that the production of excessive ROS and the activation of NLRP3 inflammasome are also mediated by thioredoxin (TRX)-interacting protein (TXNIP), i.e., the endogenous suppressor of the TRX system [82]. The TRX system is the main inhibitor of the production of cellular mercaptan and exerts anti-inflammatory and antioxidative effects. In addition, TXNIPs can act as crucial pro-inflammatory mediators that link oxidative stress and inflammation towards the activation of NLRP3 inflammasome [83,84]. In fact, TXNIP also serves as primary molecular signaling site in response to inflammation and ER stress [85]. Furthermore, following molecular and cellular stress—the release of lysosomal cysteine protease cathepsin into the cytoplasm after lysosomal membrane rupture also mediates the activation of NLRP3 inflammasome [86]—this was observed after the cleavage of the NLRP3 receptor, which is associated with inhibitory protein domains [86]. Finally, following an ischemic stroke—hypoxia, vascular obstruction, and cerebral parenchyma injury/ischemia lead to anaerobic glycolysis—this leads to the excessive accumulation of hydrogen ions (H^+^) and lactic acid, which induces acidosis and subsequent NLRP3 activation [87]. Figure 3 describes the brief mechanism of NLRP3 activation and action and their role in the molecular mechanism of AIS and COVID-19-mediated ischemic stroke.

### 4.2. NLRP3 Inflammasome and Ischemic Stroke

As previously mentioned, inflammasome can serve as a mediator of the activation of caspase-1, whereby the activated or cleaved caspase-1 may lead to inflammation-related cell death or programmed cell death, called pyroptosis [88]. Pyroptosis is characterized by gasdermin-D-mediated cell death (GSDMD) through a complete mitochondrion ballooning and destruction [89]. Caspase-1 mediates the cleavage of GSDMD and triggers GSDMD-N domain oligomerization, which results in the formation of membrane pores. On the other hand, caspase-1 regulates the protein cleaving and pruning of pro-IL-1β and pro-IL-18 into functional pro-inflammatory cytokines, i.e., IL-1β and IL-18, respectively, and releases them into the extracellular space [72] through membrane pores generated by GDFMD [89]. A previous study proposed that GSDMD may serve as a plausible therapeutic target to inhibit caspase-1 activity [90]. Moreover, caspase-1 activation can also be inhibited through the administration of caspase-12, thereby suppressing the activation of NLRP3 inflammasome [91].

Additionally, most metabolic changes and stress take place in intra- and extracellular environments (i.e., reduced K^+^ inflow, increased intracellular Ca^2+^, reduced ATP production, and heightened mitochondrion-mediated ROS release) following an ischemic stroke and cannot be eliminated through normal homeostasis and/or leukocyte recruitment to the insult sites. Therefore, NLRP3 inflammasome is activated and leads to the maturation of pro-caspase-1 into caspase-1, which results in the further release of pro-inflammatory molecules, causing pyroptosis (Figure 3). However, one animal study has shown that specific pyrophosphate inhibitors, such as Vx765, act as molecular blockers for NLRP3 inflammasome, thereby offering potential therapeutic value for ischemic stroke recovery and rehabilitation [92]. Furthermore, hypothermia also enables the inhibition of pyroptosis, thereby reducing pyroptosis after ischemic events. Finally, NLRP3 inhibitors, such as MCC950, have been reported to successfully inhibit the activation of NLRP3 after ischemic stroke and alleviate cerebral infarct size, pyroptosis and neuronal apoptosis, and neurological dysfunction [93]. Therefore, there is enough evidence to suggest that targeting the molecular mechanisms pertaining to the role of NLRP3 inflammasome is a beneficial approach to help brain repair following ischemic stroke.

### 4.3. COVID-19 Related Ischemic Stroke and NLRP3 Inflammasome

As mentioned in the previous section, the excessive production of inflammatory cytokines or a cytokine storm may lead to BBB damage, and SARS-CoV-2 may enter the brain parenchyma. In this case, neuroglial cells, such as microglial cells and astrocytes, are the first responders; they are highly sensitive to cytokine storms. They may receive the inflammatory signal from ECs to further promote the expression of pro-inflammatory genes, leading to neuroinflammation and neurodegeneration [3,94]. Notably, it has been suggested that cytokines (i.e., IL-6) and NLRP3 inflammasome are the main immune components that mediate the cascade of cytokine storms and immune response during viral invasion [95,96]. Hence, it is plausible to deduce that NLRP3 inflammasome is somewhat involved in COVID-19-related cytokine storms.

Interestingly, SARS-CoV-2 and its components can mediate NLRP3 inflammasome activation through the activation of P2X7R and heightened extracellular ATP levels [95]. This is due to the fact that neuro-glial cells have high P2X7R expression. As explained in the previous section, the downregulation of ACE2 mediates the activation and expression of P2X7R following angiotensin II increment; hence, it is reported that this, in turn, increases the activation of NLRP3 inflammasome, leading to neuro-glial invasion and neuroinflammation [95]. Furthermore, SARS-CoV-2 and its protein components can also bind to mannan-binding lectin (MBL), which in turn induces the activation of a coagulation cascade through MBL-associated serine protease 2 (MASP-2) complex, elevating the activation of NLRP3 inflammasome [97]. Following NLRP3 inflammasome action is the downstream release of DAMPs (i.e., the excess release of nuclear protein high-mobility group box 1 (HMGB1]) through GSDMD, as demonstrated by the hyperinflammation during viral infection and/or COVID-19 [98]. Taken together, there is encouraging evidence for the proposition that targeting the immune cascade and molecular signaling pertaining to NLRP3-inflammasome-mediated inflammation and hypercoagulability may provide fresher avenue into a therapeutic strategy for brain repair following COVID-19 ischemic stroke and future post-acute and/or long COVID-19 syndrome. Further evidence pertaining to the positive outcome of NLRP3 inflammasome downregulation and molecular modulation prior to brain repair (i.e., neuro-gliogenesis and/or synaptogenesis) is discussed in the following section.

## 5. Emerging Neurorehabilitation Strategies for COVID-19-Related Ischemic Stroke

It has been described elsewhere that spontaneous and rapid biological recovery (i.e., molecular, and cellular behavioral response towards biological events) takes place immediately (i.e., during the first few weeks and months) following cerebral ischemia (or post-stroke) [99]. This recovery is mainly coordinated by molecular and cellular mechanisms characterized by the promotion of post-stroke neuroplasticity, brain repair, and reorganization, including lesion and non-lesion areas of the brain parenchyma [100,101]. Preclinical studies spatially characterized post-stroke neuroplasticity as the formation of new synapses and connections (or synaptogenesis), axonal sprouting, dendritic branching that aids in changes in synaptic compensation, strength, and neuro-gliogenesis by contralateral or ipsilateral cortical regions [102,103]. This recovery response was comparable in both pre-clinical (animal) and clinical (human) studies [101].

Previous studies have characterized the mechanism of post-stroke neuroplasticity and brain repair into four major and overlapping phases, including inflammatory cascade and resolution of injury, diaschisis (i.e., damage-mediated remote effects on distal structurally and functionally connected brain parenchyma), tissue repair, and neuro-glial compensation [104,105]. However, the uncontrolled inflammatory reaction following ischemia can further impact the tissue damage (for several days to months), thereby expanding the lesion and neurological damage [106]. Therefore, further understanding of the molecular and cellular processes responsible for post-ischemic stroke neuroplasticity and brain repair (remodeling) may give a beneficial insight into brain structural and functional recovery after COVID-19 ischemic stroke and prevention for post-acute/long COVID-19 syndrome. Hence, in this section, we describe and propose converging approaches targeting these molecular and cellular mechanisms, focusing on the mediating role of the ACE2/Ang-(1–7)/MasR axis and NLRP3 inflammasome to expand the understanding of COVID-19 related ischemic stroke rehabilitation (i.e., neuroplasticity and brain repair).

### 5.1. Mediating Roles of RAS and the ACE2/Ang-(1–7)/MasR Axis in Ischemic Stroke Rehabilitation

The brain’s glial cells (i.e., astroglia) are recognized for their role in neuroinflammation and oxidative stress. Since AT1R in RAS is important for pro-inflammatory agents and oxidative stress, previous studies established the neuroglial cell interaction with brain RAS in the pathomechanism of several neurological diseases [107,108] as well as molecular therapeutic strategies for potential brain repair [109]. Moreover, the crosstalk between RAS components, such as AT1R, mediated the increment of pro-inflammatory agents and ROS, leading to Ang II activation. This, in turn, triggered neuropathological complications. On the other hand, AT1R also mediated the crosstalk with multiple receptors, such as GPCR and MasR, thereby playing a role in neuroprotection [110].

Various studies have reported that RAS modulators have the potential to improve post-stroke structural and functional recovery [111,112]. Moreover, pre-clinical studies have also demonstrated that the modulation of the ACE2/Ang-(1–7)/MasR axis may promote neuroprotection against ischemic stroke [113,114,115]. Many studies have also reported the pragmatic impact of RAS and ACE2/Ang-(1–7)/MasR axis modulators on neuroplasticity and brain repair, which also includes AT1R blockers (ARBs) and enzyme inhibitors (i.e., renin and ACE inhibitors, and AT2R agonists) [116]. Table 1 summarizes several emerging, positive RAS modulator roles in molecular neuroplasticity and brain repair mechanisms.

Moreover, a recent study reported improvements in memory in older adults with hippocampal white matter hyperintensities (i.e., a manifestation of cerebral small vessel disease related to ischemic stroke) after treatment with ARBs [131]. As a matter of fact, another study has suggested that ARBs (e.g., candesartan, telmisartan, and valsartan) can cross the BBB, thereby helping in the preservation of neurocognitive function, and reduce white-matter hyperintensity volume [132]. Apart from RAS modulators, the mediating role of the ACE2/Ang-(1–7)/MasR axis may new open windows on neuroplasticity and the molecular mechanism of brain repair following ischemic stroke. The evidence discussed thus far supports the potential neuroprotective role of the Ang-(1-7)/MasR axis against AIS and COVID-19-related ischemic stroke. Previous studies have identified the beneficial effect of Ang-(1-7)/MasR, whereby the administration of Ang-(1-7) mediated by MasR in an endothelin-induced MCAO rat model reduced the infarct size and neurological disease manifestation 72 h post-stroke [51]. Furthermore, several studies have shown that the intracerebral infusion of Ang-(1-7) into a permanent MCAO (pMCAO) rat model ameliorated the infarct size and volume and improved neurological dysfunction 24 h [113] and 4 weeks [114] after insults.

Therefore, it has been suggested that Ang-(1-7) mediated by MasR may exert an antioxidative effect, reducing and inhibiting pro-inflammatory cytokines, cyclooxygenase-1 (COX-1), and NF-κB activity, respectively in peri-infarct areas [113,114]. Moreover, the Ang-(1-7)/MasR axis also mediated the stimulation of endothelial nitric oxide synthase (eNOS) and endothelial cell proliferation, thereby improving cerebral capillary density [114]. Furthermore, the Ang-(1-7)/MasR axis also modulated the reduction in NADPH oxidase (NOX) and the subsequent reduction in ROS, thereby protecting the cerebral parenchyma from cellular swelling and pyroptosis following ischemic injury [133]. Thus, there is enough evidence to postulate that the Ang-(1-7)/MasR axis may offer neuroprotective, anti-inflammatory, and anti-oxidative potentials against adverse the effects of post-ischemic stroke (with or without COVID-19) and aid in brain repair and recovery [51,113,114].

### 5.2. Mediating Role of NLRP3 Inflammasome in Ischemic Stroke Rehabilitation and Brain Repair

As highlighted earlier, the NLRP3 inflammasome is a crucial mediator of inflammation and neuro-glial damage after AIS and COVID-19-mediated ischemic stroke. Hence, a study on the various components of the NLRP3 inflammasome molecular pathway may give beneficial findings on stroke recovery and brain repair. There are several novel approaches that can be considered for therapeutic treatment targeting molecular gene expression and products to influence NLRP3 inflammasome activation and action. Multiple promising pre-clinical animal studies have utilized typical drugs (modulators) targeting various neuro-glial cells and molecular pathways to promote brain repair and recovery. These are summarized in Table 2.

Apart from the modulators listed in Table 2, research has also suggested that treatment with gene expression products using MCC950 showed a positive outcome. MCC950 may improve BBB integrity and reduce BBB damage to ameliorate post-ischemic neuro-glial cells death and improve neurological function [154]. This is the case because MCC950 treatment inhibits the activation and effects of the NLRP3 inflammasome [155]. Finally, animal studies have also shown that the administration of ethyl methyl ketone (i.e., voltage-gated K^+^ channel suppressor) can help to prevent NLRP3 receptor activation [156]. Thus, it seems that the inhibition of NLRP3 inflammasome activation and effects may point towards a better understanding of the molecular mechanism that can be further explored in post-ischemic stroke (with or without COVID-19) brain repair.

## 6. Propositions and Future Prospects

**Figure 4:**
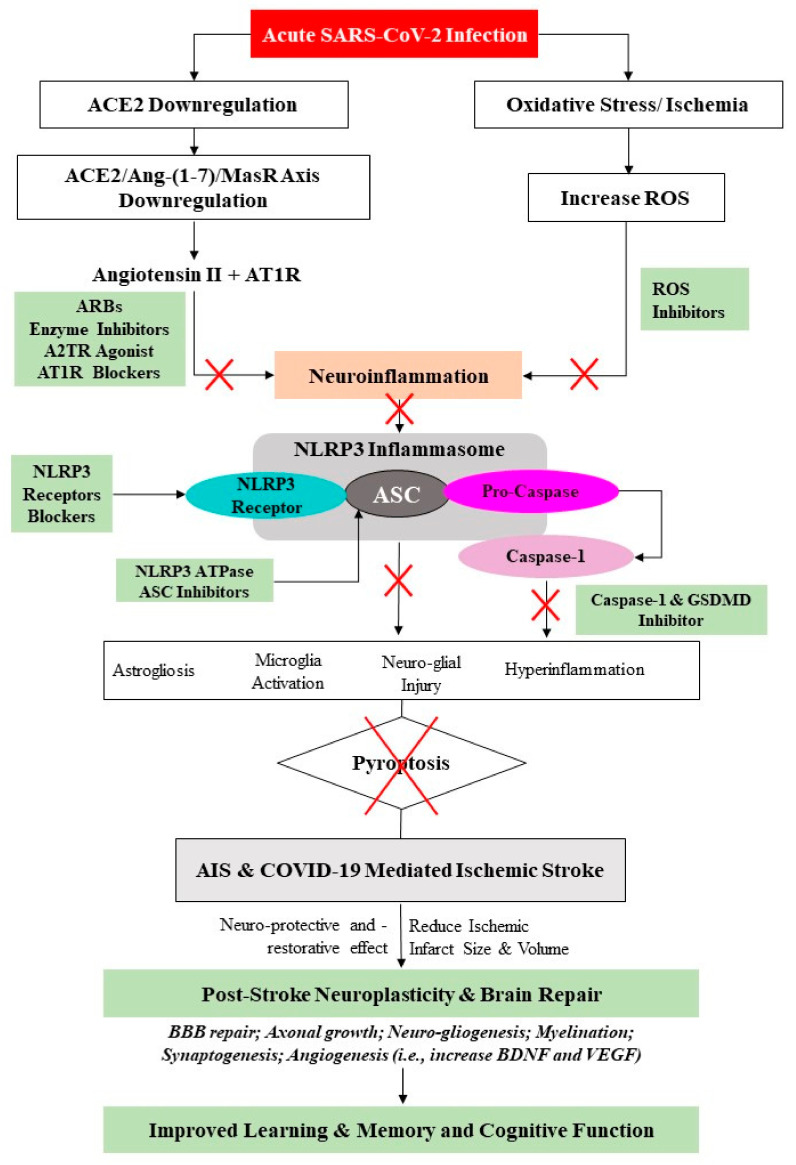
Proposed mediating role of ACE2/Ang-(1-7)/MasR axis and NLRP3 inflammasome in AIS and COVID-19-mediated ischemic stroke and the prospects of these neuroinflammation mediators for brain repair and in secondary prevention strategies against AIS in stroke rehabilitation.

Despite the rapid growth of studies pertaining to the underlying pathophysiological mechanism of COVID-19-mediated ischemic stroke, the exact mechanism remains elusive. However, numerous reports have indicated a significant increase in the incidence of COVID-19-mediated ischemic stroke, which were attributed to hyper-inflammation (i.e., cytokine storm) and hypercoagulation. These, in turn, cause remote effects and/or ischemic lesions on distant but structurally and functionally connected brain areas [157]. Moreover, central to the underlying neurobiology of stroke recovery in COVID-19 infection is reduced ACE2 expression, which is known to lead to thrombo-inflammation and ACE2/Ang-(1-7)/MasR axis inhibition. Furthermore, after AIS, the activated NLRP3 inflammasome may heighten the production of numerous pro-inflammatory cytokines, mediating neuro-glial cell dysfunction, ultimately leading to nerve-cell death. Therefore, in this narrative review, several potential neuroprotective therapies targeting the molecular mechanism of the aforementioned mediators were proposed. These may help to inform clinical practitioners as to rehabilitation strategies to improve brain reorganization, such as neuro-gliogenesis and synaptogenesis, as well as secondary prevention among AIS patients with or without COVID-19. Figure 4 summarizes the proposed mediating role of the ACE2/Ang-(1-7)/MasR axis and the NLRP3 inflammasome in COVID-19-mediated AIS and the prospects of these neuroinflammation mediators for brain repair and in secondary prevention strategies against AIS in stroke rehabilitation.

Additionally, recent studies have suggested that alongside mass vaccination, the restoration of cytokine balance (i.e., through recovering pro-inflammatory agent trans-signalling using neutralizing buffer system) may also promote a novel approach to the physiological treatment for COVID-19 patients. This would provide a further guide for clinical strategies to ameliorate the adverse effects of the infection i.e., the post-acute/long COVID-19 syndrome, in patients with or without COVID-19-mediated ischemic stroke. This is emphasized by the fact that cytokine restoration may interfere with and inhibit SARS-CoV-2 entry and infection, which in turn inhibits hyper-inflammation and hyper-coagulopathy [158,159]. Furthermore, this effort may also help to maintain the ACE2/Ang-(1-7)/MasR axis and reduce NLRP3 inflammasome activation, which would favor brain repair and secondary prevention efforts. Finally, further neuroimaging (from structural to functional) studies are required to study the network effect distant to the ischemic lesion prior to the proposed therapeutic strategies to further confirm the outcomes and to enhance the understanding of the complex molecular networks in human brain reorganization during repair to and recovery from an ischemic stroke-induced deficit.

## 7. Conclusions

This narrative review highlights the need for a more complete understanding of the ACE2/Ang-(1-7)/MasR axis and the NLRP3 inflammasome to elicit neuroprotection, with or without the setting of COVID-19-mediated ischemic stroke. In the context of COVID-19 infection and AIS, these mediators serve as plausible putative molecular targets for potential therapeutic avenues to enhance neuroplasticity and functional recovery in stroke rehabilitation and secondary prevention strategies.

## Figures and Tables

**Table 1:** Positive role of RAS modulators in molecular neuroplasticity and functional brain repair.

Ras Modulators	Impact On Neuroplasticity and Functional Brain Repair
**(A) ARBs**	
Telmisartan	Improved memory in a cerebral ischemic animal model (i.e., rats) [117]. Improved spatial learning and memory in SP-SHR [118]. Improved diabetes-induced cognitive decline by reducing diabetes-induced increases in BBB permeability through PPAR activation [119].
Losartan	Attenuated astrogliosis and normalizes the activation levels of AT1R and AT4R [120]. Improved learning and memory in AD mice model with APP [121].
Valsartan	Possess anti-stress effects and helped improve learning and memory in a cognitively impaired animal model with chronic stress and glucocorticoid [121,122].
Candesartan	Possess neurorestorative effect and help reduce infarct size and functional recovery in tMCAO rat stroke models [123]. Helped increase the expression of BDNF, synaptophysin, VEGF, angiopoetin-1, and TrkB to promote angiogenesis [123,124].
**(B) Enzyme Inhibitors**	
Renin Inhibitor	Aliskiren—inhibited the catalytic effects of renin in RAS hence blocking the AT1R [125].
ACE Inhibitor	Perindopril—improved cognitive function in an animal model with VCI and AD [124,126] and the interplay between dopaminergic and ACE neurotransmission and/or reduction of acetylcholinesterase activity. Furthermore, it helped increase cBF, reduced oxidative and nitrative stress, and reduced amyloid-β deposition [126,127]. Ramipril—reduced microglial activation in dentate gyrus and improved cognition [128].
AT2R Agonist	Improved spatial memory and prevented cognitive deficits in an animal model with AD [129]. Decreased hippocampal neurogenesis and increased cognitive impairment in an AT2R-knockout animal model [130].

ACE, angiotensin-converting enzymes; AD, Alzheimer’s disease; APP, amyloid-β precursor protein; ARBs, angiotensin I receptor (AT1R) blockers; BDNF, brain-derived neurotrophic factor; cBF, cerebral blood flow; RAS, renin angiotensin system; SP-SHR, stroke-prone spontaneously hypertensive rats, tMCAO, transient middle cerebral artery occlusion; TrkB, tyrosine kinase B; VCI, vascular cognitive impairment; VEGF, vascular endothelial growth factor.

**Table 2:** NLRP3 inflammasome modulators for ischemic stroke recovery.

Pre-Clinical Models	Target Cells	Drugs/ Modulators	Molecular Impact on Neuroplasticity
Mice BMDMs	Macrophages	Parthenolide	Direct inhibition of caspase-1, thereby inhibiting inflammasome in macrophages [134].
		MNS	Interfered with NLRP3-induced ASC speck formation and inhibited NLRP3 ATPase activity, reducing inflammatory response [135].
		O3FA	Stopped NLRP3-inflammasome-mediated inflammatory response [136].
		Curcumin	Suppressed NLRP3 inflammasome activation by inhibiting K^+^ outflow, inhibited caspase-1 and IL-1β activation [137].
		A151	Inhibited the expression of pyroptosis-associated proteins, including GSDMD, caspase-1, IL-1β, and IL-18 [138].
MCAO Mice/Rats	Neurons Microglial Astrocytes	EPA	Inhibited NLRP3 activation and stopped apoptosis-mediated acute cerebral infarction [139].
		Sinomenine	Inhibited NLRP3 inflammasome via AMPK pathway, reduced post-stroke cerebral infarction, edema, neuronal apoptosis, and neurological impairment [140].
		Artigenin	Inhibited ischemic-stroke-mediated NLRP3 inflammasome activation and secretion of IL-1β and IL-18 [141].
		IVIG	Alleviated infarct volume and neuronal cell death; improved brain function; increased anti-apoptotic protein BCL-2 expression; inhibited NLRP3 by blocking binding between NACHT domain and ATP in NLRP3 receptor; inhibited proinflammatory cytokine expression [142].
		Umbelliferon	Alleviated TXNIP expression and NLRP3 inflammasome activation [143].
		JQ1	Inhibited the production of pro-inflammatory agents, NF-κB, NLRP3 inflammasome, and caspse-1 activation; alleviated infarct size, improved neural function, and protected the brain against ischemic insults [144].
		Meisoindigo	Reduced post-stroke ischemic injury by inhibiting NF-κB pathway [145].
		GDLs	Downregulated NF-κB pathways, reducing pro-inflammatory cytokine release, astrocyte activation, and platelet aggregation [146].
tMCAO Mice/Rats	Neurons Microglial	Minocyclin	Inhibited microglia and NLRP3 inflammasome activation; inhibited the release of pro-inflammatory cytokines; reduced infarct volume and improved neurological function after ischemic stroke [147].
		Chrysophanol	Inhibited NLRP3, caspase-1, and IL-1β expression and helped against cerebral ischemia [148].
		NM	A serine protease inhibitor that inhibits the activation of pro-inflammatory mediators; interfered in NF-κB pathways to inhibit NLRP3 activation [149].
Others	Neurons Microglial Astrocytes	Isoliquiritigenin	Inhibited ROS- and NF-κB-mediated NLRP3 activation [150].
		Corylin	Inhibited NLRP3 inflammasome activation [151].
		Nicorandil	An ATP-sensitive K^+^ channel opener that inhibits NLRP3 inflammasome activation [152].
		Probenecid	A pannexin-1 inhibitor—inhibited caspse-1, NLRP3, aquaporin 4, and IL-1β activation and release. Promoted neuroprotection against ischemic insults [153].

Notes: A151 is a synthetic oligodeoxynucleotide containing multiple distal -TTAGGG- sequences. JQI is a bromodomain-containing protein 4 (BRD4) inhibitor. AMPK, adenosine monophosphate-activated protein kinase; ASC, apoptosis-associated speck-like protein with a CARD; ATP, adenosine triphosphate; BCL-1, B-cell lymphoma 2; BMDMs, EPA, eicosapentaenoic acids; bone marrow–derived macrophages; GDLs, ginkgo diterpene lactones; IL-1β, interleukin 1 beta; IVIG, intravenous immunoglobulin; MCAO, middle cerebral artery occlusion; MNS, 3,4-methylenedioxy-β-nitro styrene; NACHT, NAIP, CIITA, HETE-E, and TP1 domains; NF-κB, nuclear factor kappa B; NLRP3, nucleotide-binding oligomerization domain (NOD)-like receptor (NLR) family pyrin domain-containing 3; NM, nafamostat mesilate; O3FA, omega-3 fatty acids; ROS, reactive oxygen species.

## Data Availability

Not applicable.
